# Cortico-Striato-Thalamo-Cortical Circuitry, Working Memory, and Obsessive–Compulsive Disorder

**DOI:** 10.3389/fpsyt.2016.00078

**Published:** 2016-05-04

**Authors:** Baojuan Li, Maria Mody

**Affiliations:** ^1^Department of Radiology, Athinoula A. Martinos Center for Biomedical Imaging, Massachusetts General Hospital, Harvard Medical School, Charlestown, MA, USA; ^2^School of Biomedical Engineering, Fourth Military Medical University, Xi’an, China

**Keywords:** obsessive–compulsive disorder, brain connectivity, frontal, striatal, thalamic circuits

Obsessive–compulsive disorder (OCD) is characterized by persistent anxious thoughts (obsessions) and repeated rituals (compulsion) (1, 2). The symptoms point to dysfunctions in inhibitory control, implicating the dorsal anterior cingulate cortex (dACC) as part of a wider cortico-striato-thalamo-cortical (CSTC) circuitry based on findings from resting-state and task-dependent paradigms.

The study by Diwadkar and colleagues (3) represents an important advance in research with patients with OCD. The authors assessed interactions between brain regions activated during a task not involving conflict monitoring. The approach helped to reveal a general network-based dysfunction at the core of OCD. Unlike resting-state functional connectivity analysis, the authors examined dysfunctional connectivity in OCD during performance of a working memory task. Although resting-state connectivity analysis involves the interactions between endogenous activities of different brain regions, task-based connectivity provides specific information on how distributed regions in a network cooperate in doing a cognitive task. Understanding such mechanisms is crucial, especially when specific cognitive processes are known to be implicated. In their study, Diwadkar et al. collected functional magnetic resonance imaging (fMRI) data in 18 participants with OCD and 27 healthy controls (HC) during a verbal *n*-back task. Although both groups demonstrated increased activation of CSTC circuits with increased memory load, the OCD group exhibited hyperactivation of the parietal lobe, mid frontal gyrus, dorsal prefrontal cortex, and dACC at both low (1-Back) and high (2-Back) levels of memory load compared to that of HC. This memory load-related hyperactivation has been proposed as an intermediate phenotype for OCD (4). Psychophysiological interaction (PPI) analysis targeting the dACC (given its role in control functions) revealed significantly greater dACC modulation of the parietal lobe, mid frontal gyrus, basal ganglia, and dorsal prefrontal cortex in the OCD group than in the controls, though dACC effects in OCD have been equivocal (5). In keeping with Menzies and colleagues (6), a revised model for OCD may be warranted, one that extends beyond a CSTC circuity to include dorsolateral prefrontal and parietal regions and implicates dysfunctional activation in tasks involving working memory.

Regardless, the findings in this study are consistent with earlier research on pediatric patients which found elevated dACC connectivity during performance monitoring (7). It also extends findings from previous studies that have reported hyperactivation or hyperconnectivity of regions in the CSTC circuits in OCD subjects during an “incentive flanker task” (8) and at rest (9–11). Using a seed-based functional connectivity analysis method, Harrison et al. (9) revealed increased resting-state ventral corticostriatal functional connectivity in OCD. Even healthy first-degree relatives of the OCD patients demonstrated increased resting-state functional connectivity of regions in the CSTC circuits (11). In a separate study, Kang et al. (12) found hypoactivation of the cingulate cortex and basal ganglia during a response inhibition task in their OCD group, but heightened resting-state striatal–cortical connectivity compared to the controls. Evidence from animal studies also confirms the involvement of this circuit in OCD. Using an optogenetic technique, Ahmari et al. (13) found increased grooming (OCD-like behaviors) in mice after repeated stimulation of the CSTC circuits (13). However, there remain some inconsistencies in this field. In a recent study, resting-state functional connectivity of the limbic CSTC loop was found to be decreased in unmedicated patients with OCD (14). The results differ from an earlier study, which reported increased resting-state distant and local connectivity of the CSCT circuits (10).

The study by Diwadkar et al. represents one of the few attempts to investigate direct connectivity in OCD. The majority of brain connectivity studies have focused on undirected functional connectivity that refers to temporal correlation between the time series of different brain regions (15). The PPI approach used in the study is a simple way to examine effective connectivity (15). The results, using dACC as the seed region, support those of Schlosser et al. who found elevated modulatory effects of the Stroop color-word task on dACC to DLPFC effective connectivity in the OCD patient group (16).

Elucidating the neural basis of OCD is of great importance to the treatment of this disorder. As more evidence accumulates to support the involvement of the CSTC circuits in the pathophysiology of OCD, researchers have started to develop treatments that target regions of the CSTC circuitry. For patients with OCD who do not respond effectively to traditional pharmacotherapy or cognitive–behavioral therapy, it has been shown that deep-brain stimulation could be another treatment alternative. In patients with refractory OCD, the stimulation of the nucleus accumbens effectively reduced the severity of OCD (17). Repetitive transcranial magnetic stimulation of the dorsomedial prefrontal cortex (dmPFC) has also yielded positive results in treatment-resistant patients. Dunlop et al. (18) found that 50% of the patients in their study responded to the treatment, and the efficacy of the treatment was correlated with the decrease in functional connectivity between the dmPFC and ventral striatum (18). The results by Diwadkar and colleagues (3) suggest a role for an extended CSTC circuitry in the neurobiology of OCD, worthy of further exploration.

## Author Contributions

All authors listed have made substantial, direct, and intellectual contribution to the work and approved it for publication.

## Conflict of Interest Statement

The authors declare that the research was conducted in the absence of any commercial or financial relationships that could be construed as a potential conflict of interest.
